# Insights into structure, codon usage, repeats, and RNA editing of the complete mitochondrial genome of *Perilla frutescens* (Lamiaceae)

**DOI:** 10.1038/s41598-024-64509-3

**Published:** 2024-06-17

**Authors:** Ru Wang, Yongjian Luo, Zheng Lan, Daoshou Qiu

**Affiliations:** 1 Key Laboratory of Crops Genetics and Improvement of Guangdong Province, Crops Research Institute, Guangdong Academy of Agricultural Sciences, Guangzhou, 510640 China; 2 Hubei Minzu University, School of Forestry and Horticulture, Enshi, 445000 China; 3 Heilongjiang Bayi Agricultural University, Daqing, 163319 China

**Keywords:** Lamioideae, Sequence characteristics, Phylogenetic analysis, Mitochondrion, RSCU, RNA editing, Biodiversity, Plant biotechnology, Plant breeding, Plant evolution, Plant genetics, Genome informatics, Mitochondrial genome

## Abstract

*Perilla frutescens* (L.) Britton, a member of the Lamiaceae family, stands out as a versatile plant highly valued for its unique aroma and medicinal properties. Additionally, *P. frutescens* seeds are rich in Îś-linolenic acid, holding substantial economic importance. While the nuclear and chloroplast genomes of *P. frutescens* have already been documented, the complete mitochondrial genome sequence remains unreported. To this end, the sequencing, annotation, and assembly of the entire Mitochondrial genome of *P. frutescens* were hereby conducted using a combination of Illumina and PacBio data. The assembled *P. frutescens* mitochondrial genome spanned 299,551 bp and exhibited a typical circular structure, involving a GC content of 45.23%. Within the genome, a total of 59 unique genes were identified, encompassing 37 protein-coding genes, 20 tRNA genes, and 2 rRNA genes. Additionally, 18 introns were observed in 8 protein-coding genes. Notably, the codons of the *P. frutescens* mitochondrial genome displayed a notable A/T bias. The analysis also revealed 293 dispersed repeat sequences, 77 simple sequence repeats (SSRs), and 6 tandem repeat sequences. Moreover, RNA editing sites preferentially produced leucine at amino acid editing sites. Furthermore, 70 sequence fragments (12,680 bp) having been transferred from the chloroplast to the mitochondrial genome were identified, accounting for 4.23% of the entire mitochondrial genome. Phylogenetic analysis indicated that among Lamiaceae plants, *P. frutescens* is most closely related to *Salvia* *miltiorrhiza* and *Platostoma chinense*. Meanwhile, inter-species Ka/Ks results suggested that Ka/Ks $<1$ for 28 PCGs, indicating that these genes were evolving under purifying selection. Overall, this study enriches the mitochondrial genome data for *P. frutescens* and forges a theoretical foundation for future molecular breeding research.

## Introduction

The mitochondrial genome is relatively small, highly conserved, and densely packed with genes, while also containing highly variable non-coding regions? In plant research, in addition to its role in energy metabolism, the mitochondrial genome generally affects plant fertility, also playing an important role in plant development and stress resistance^1^. Comparative analyses of mitochondrial genome sequences, structures, and functions reveal the complexity of plant mitochondrial genomes, providing a basis for deciphering their classification and evolution, and facilitating the exploration of cytoplasmic male sterility mechanisms^2,3^. However, research on complete plant mitochondrial genomes lags far behind that of complete plastid genomes. The NCBI database hosts nearly asting with only 673 complete plant mitochondrial genomes^4^, which is attributed 13,000 complete plastid genomes, contrasting with only 673 complete plant mitochondrial genomes^4^, which is attributed to the complex structure of plant Mitochondria. They are challenging to purify and often face interference from chloroplasts and other organelles, complicating genome assembly. In addition, significant differences are observed in the structure and content of plant mitochondrial genomes, as well as in nucleotide substitution rates and repeat sequences^3,5^. Therefore, a complete description of the plant mitochondrial genome remains a bottleneck in evolutionary biology, yet most plant systematic studies focus on nuclear and plastid genomes^6^. For example, while the nuclear and plastid genomes of* Perilla frutescens * have been sequenced and assembled, its mitochondrial genome remains unknown^7–9^. With the development of high-throughput sequencing technology and the rise of next-generation systems genomics, many software programs applicable to mitochondrial genome sequencing and assembly, such as GetOrganelle^10^, Mitofiner^11^, GSAT^12^, and PMAT^13^ (https://github.com/bichangwei/PMAT), have been developed, making mtochondrial genome sequencing and assembly more accurate and efficient^14^.

Besides, in land plants, mitochondrial genomes typically contain exogenous genes or fragments due to horizontal gene transfer or intracellular gene transfer^15,16^. As the mitochondrial genome undergoes continuous recombination, this sequence transfer between genomes also occurs^17^. This can result in cytoplasmic male sterility (CMS), where plants cannot produce viable pollen grains due to a mismatch between the nuclear and cytoplasmic genomes. However, female fertility is maintained, as observed in Brassica, rice, and other plants^17–19^. Additionally, CMS is often inherited maternally and is commonly associated with abnormal open reading frames (ORFs) and RNA editing in the mitochondrial genome^20^. These ORFs are often found near functional mitochondrial genes and are co-transcribed with them, affecting the mitochondrial function^21^. CMS-based hybridization techniques have been utilized to breed offspring with significantly improved yield, stress resistance, and adaptability, making this approach a promising way to maintain crop productivity^22^. Therefore, mitochondrial genomes are a valuable source of genetic information for plant systematics and necessary cellular process research. They have significant implications for species evolution research, species identification, and genetic transformation^23^.

*Perilla frutescens* (L.) Britt. belongs to the Lamiaceae family, and is an annual upright herb. It is renowned as a traditional dual-purpose medicinal and edible plant, prized for its distinct aroma^24^. Notably, its leaves, stems, and fruits all possess medicinal properties. *P. frutescens* is widely cultivated in China and Southeast Asia, ranging from Indonesia in the south to Japan in the east^25^. Current research on *P. frutescens *mainly focuses on its pharmacological effects and chemical composition of its compound perillaldehyde^26^. Currently, 400 compounds have been isolated from *P. frutescens* leaves, including terpenoids, flavonoids, alkaloids, steroids, quinines, and phenolic compounds, all of, which hold significant potential for various applications^27–29^. People have discovered the pharmacological functions of *P. frutescens* attributed to secondary metabolites present in different parts of the plant, such as anti-allergic, anti-depressive, lipid-lowering, hepatoprotective, neuroprotective, anti-inflammatory, anti-cancer, antioxidant and antibacterial activities^30–33^. In addition, common oil crops such as soybean, rapeseed, peanut, and olive generally have an $\alpha$-linolenic acid content of less than 5$\%$^34,35^. Conversely, *P. frutescens* seeds boast a high oil content of 45–55$\%$, with a rich content of unsaturated fatty acids, accounting for more than 90$\%$ of the total oil content. Among which, $\alpha$-linolenic acid has the highest content, reaching 55 65$\%$^36^. Consequently, *P. frutescens* is considered a natural resource possessing significant economic and medicinal value. Despite being a plant of significant medicinal and economic value, the mitochondrial genome of *P. frutescens* frutescens has not yet been assembled and analyzed. This crucial step is essential for comprehending its genetic composition and realizing its full potential for diverse applications.

In this study, sequencing and annotation of the *P. frutescens* mitochondrial genome were conducted, and mitochondrial genome features, RNA editing, and codon bias were comprehensively analyzed. Besides, systematic evolutionary analysis was also carried out to provide essential background information for further understanding the genetics of this plant. Overall, this research lays the groundwork for future studies on molecular breeding strategies for *P. frutescens*.

## Results

### Mitochondrial gene organization and features of *P. frutescens*

**Figure 1:**
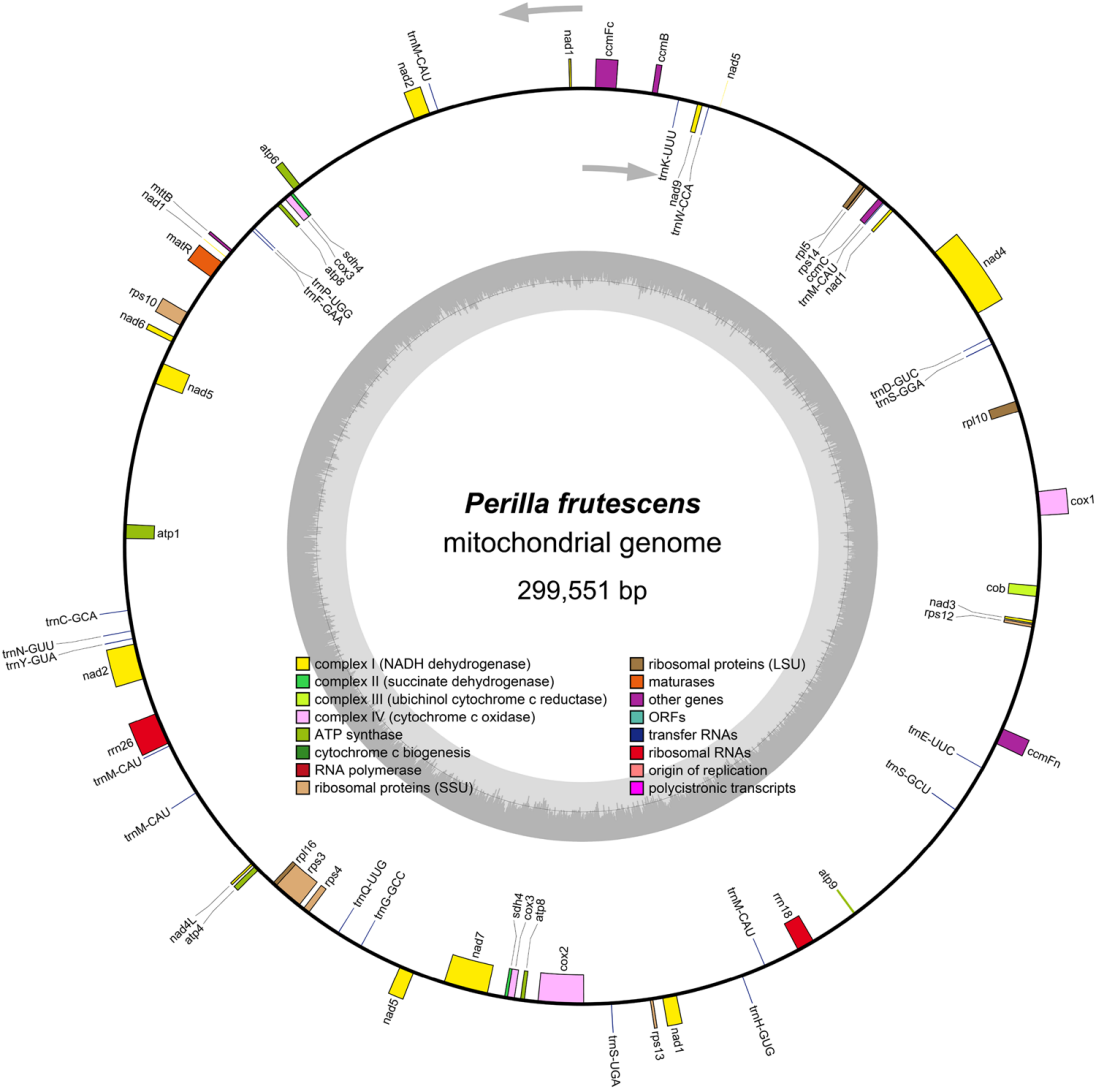
The mitogenome map of *P. frutescens*.

In this study, the mitochondrial genome was sequenced using Illumina and Pacbio sequencing platforms. A total of 248,479,765 clean reads (Q30 = 89.78$\%$) were generated from the second-generation sequencing platform, while the third-generation sequencing platform produced 7,853,744 clean reads. A total of 63,122,358,854 bases were generated. The subreads possessed an N50 value of 9597 bp and an N90 value of 4224 bp. The longest subread possessed a length of 65,388 bp. The complete circular mitochondrial DNA molecule of *P. frutescens* with a length of 299,551 bp was obtained through de novo assembly (Fig. 1). The nucleotide composition of *P. frutescens* mitochondrial DNA was 27.3$\%$ A, 27.5$\%$ T, 22.5$\%$ G, and 22.7$\%$ C, with a GC content of 45.23$\%$. Ultimately, the *P. frutescens* mitochondrial genome sequence was archived in CNGB with the accession number AA059311.1.

The mitochondrial genome of * P. frutescens* encompasses a total of 59 genes, consisting of 37 protein-coding genes (PCGs), 20 transfer RNAs (*trn*As), and 2 ribosomal RNAs (rRNAs) (refer to Table 1). The essential genes include 6 *ATP* synthase genes (*atp*1, *atp*4, *atp*6, *atp*8, and *atp*9), 9 *NAD*H dehydrogenase genes (*nad*1, *nad*2, *nad*3, *nad*4, *nad*4L, *nad*5, *nad*6, *nad*7, and *nad*9), 4 cytochrome c biogenesis genes (*ccm*B, *ccm*C, *ccm*FC, and *ccm*FN), 4 cytochrome c oxidase genes (*cox*1, *cox*2, and *cox*3), 1 Maturases gene (*mat*R), 1 membrane transport protein gene (*mtt*B), and 1 ubiquinol cytochrome c reductase gene (*cob*). The variable genes include 3 large subunits of ribosomal protein (*rpl*16, *rpl*10, *rpl*5), 6 small subunits of ribosomal protein (*rps*10, *rps*12, *rps*13, *rps*14, *rps*3, *rps*4), and 2 succinate dehydrogenases (*sdh*4). Herein, notably, the genes *atp*8, *cox*3, and sdh4 were observed in duplicate copies. Introns were found in the *nad*1, *nad*2, *nad*5 (with four introns each), *nad*4, and *nad*7 genes (with three introns each), as well as in *ccm*FC, *cox*1, *cox*2, *rps*10, and *rps*3 (each containing one intron). Furthermore, the *sdh*4 sequences were identified as pseudogenes. Among the transfer RNA genes, *trn*M-CAU was duplicated 5 times, while the remaining *trn*A genes existed as identical copies within the mitochondrial genome.

**Table 1:** Classification of genes in the *P. frutescen* mitochondrial genome.

Group of genes	Gene name
*ATP*** synthase**	*atp*1, *atp*4, *atp*6, *atp*8(2), *atp*9
Cytohrome c biogenesis	*ccm*B, *ccm*C, *ccm*Fc*, *ccm*Fn
Ubichinol cytochrome c reductase	*cob*
Cytochrome c oxidase	*cox*1*, *cox*2*, *cox*3(2)
Maturases	*mat*R
Transport membrance protein	*mtt*B
NADH dehydrogenase	*nad*1****, *nad*2****, *nad*3, *nad*4***, *nad*4L, *nad*5****, *nad*6, *nad*7***, *nad*9
Ribosomal proteins (LSU)	^#^*rpl*16, *rpl*10, *rpl*5
Ribosomal proteins (SSU)	*rps*10*, *rps*12, *rps*13, *rps*14, *rps*3*, *rps*4
Succinate dehydrogenase	^#^*sdh*4(2)
Ribosomal RNAs	*rrn*18, *rrn*26
Transfer RNAs	*trn*C-GCA, *trn*D-GUC, *trn*E-UUC, *trn*F-GAA, *trn*G-GCC, *trn*H-GUG, *trn*K-UUU, *trn*M-CAU(5), *trn*N-GUU, *trn*P-UGG, *trn*Q-UUG, *trn*S-GCU, *trn*S-GGA,*trn*S-UGA, *trn*W-CCA, *trn*Y-GUA

Notes: (*): intron number; (#): Pseudo gene; Gene (2): Number of copies of multi-copy genes.

### Repeat sequence analysis

**Figure 2:**
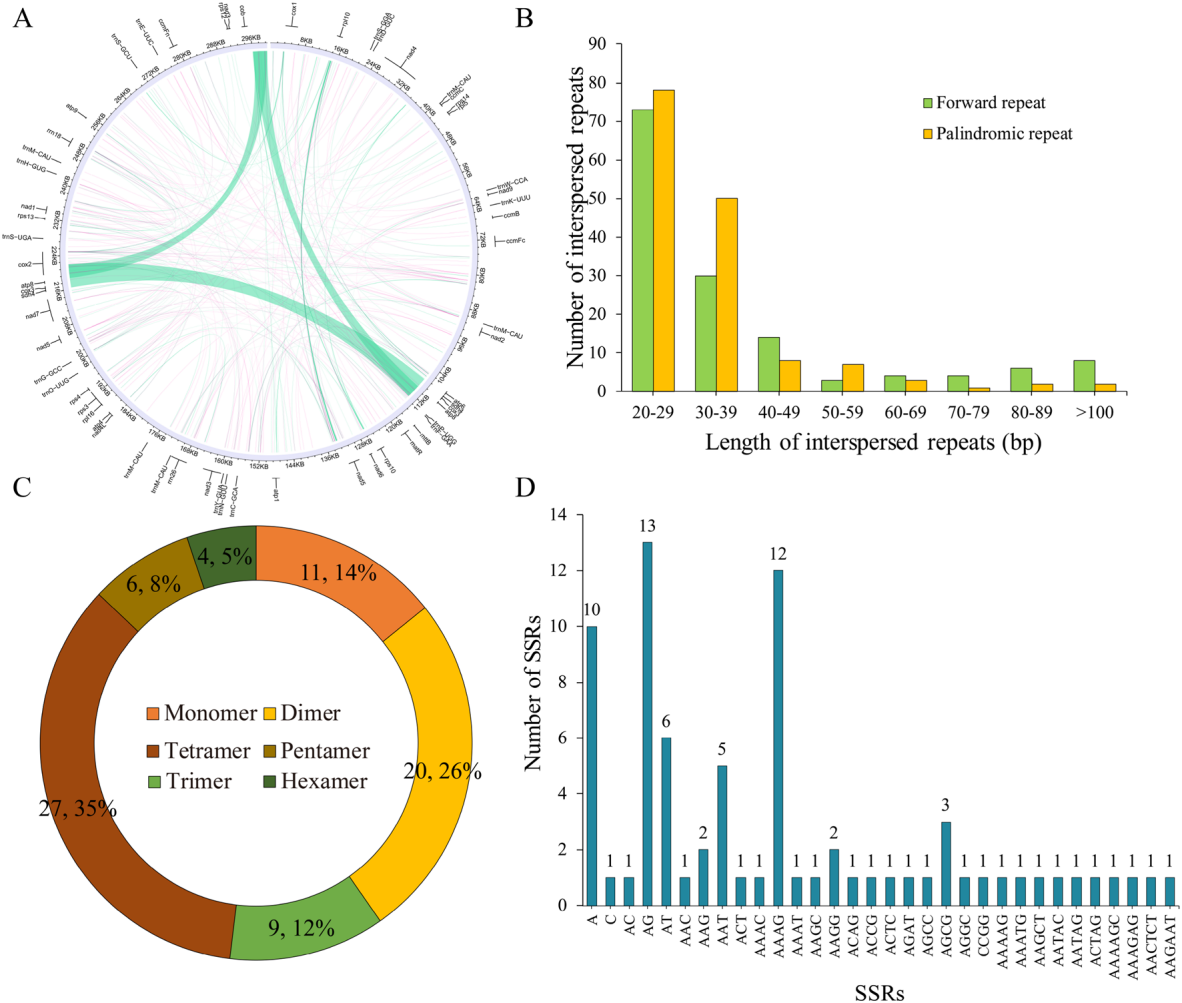
Mitochondrial genomic repeat sequences of *P. frutescen*. (**A**) Distribution of repeat sequences on the genome; (**B**) Allocation of the lengths of dispersed repeats in the *P. frutescen* mitogenome; (**C**) Statistical histogram of the *P. frutescen* mitochondrial genome with repeat sequences of different lengths; (**D**) SSR motif analysis.

In the plant mitochondrial genome, there are abundant repetitive sequences, including simple sequence repeats (SSRs), tandem repeats, and dispersed repeats^37^. In the *P. frutescens* mitochondrial genome, two different types of dispersed repeats, i.e., Forward and Palindromic, were identified, totaling 293 dispersed repeats with a length greater than or equal to 20 bp. No reverse or complementary repeat sequences were detected. The dispersed repeat sequences were visualized using the Circos software package. The results uncovered the wide distribution of these repeats in the intergenic regions of these repeats in the intergenic regions of the entire genome (Fig. 2A and Supplementary Table S1). Besides, a total of 293 dispersed repeat sequences were identified, with a combined length of 22,2651 bp, accounting for 3.76$\%$ of the total mitochondrial genome length. Among these repeats, there were 151 forward repeats and 142 palindromic repeats (Fig. 2B). The longest forward repeat sequence was 5545 bp, while the longest palindromic repeat sequence was 222 bp. Further analysis demonstrated 20–29 bp repeats as the most common type, accounting for 51.53$\%$ of the total repeat occurrences. The presence of these dispersed repeat sequences might be related to the structure and function of the mitochondrial genome.

Microsatellites (simple repeat sequences, SSRs) are typically tandem sequences of up to 6 base pairs in eukaryotic genomes^38^. In this investigation, a total of 77 SSRs were identified in the mitogenome of *P. frutescens* (Fig. 2C), dominated by tetrameric repeats, accounting for 27.35$\%$ (27) of the total count, followed by dimeric repeats (20), monomeric repeats (11), trimeric repeats (9), pentameric repeats (6), and hexameric repeats (5). Adenine (A) repeats took up the highest proportion (90.91$\%$) within the monomeric SSRs, while AG repeats occupied the highest proportion among dimeric repeats (65.00$\%$) (Fig. 2D). None of the repeat types exhibited sequences with a repeat length exceeding 20 base pairs. These widely distributed SSRs provided rich potential molecular markers for the identification and genetic research of * P. frutescens* plants.

Additionally, tandem repeats, also known as satellite DNA, are characterized by repeat lengths of 1–200 bases with varying numbers of repeats^39^. Herein, 6 tandem repeat sequences with lengths between 34 and 60 base pairs were identified in the *P. frutescens* mitochondrial genome, all of which were present in the intergenic regions (Supplementary Table S2).

### Homologous sequence analysis of organellar genomes

**Figure 3:**
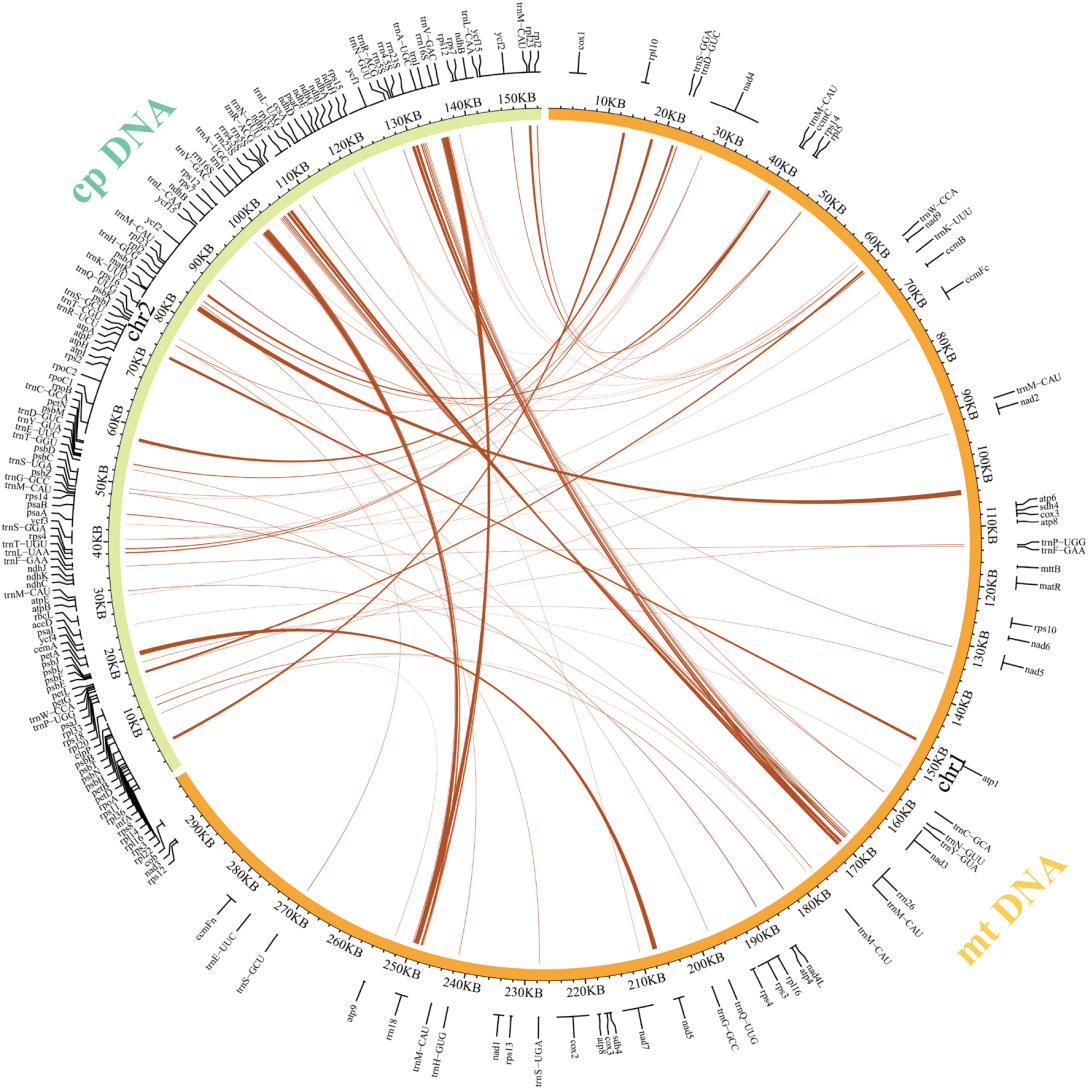
Fragments transferred from chloroplasts to mitochondrial in the *P. frutescen* genome.

The mitochondrial genome of *P. frutescens* (299,551 bp) is approximately 1.96 times larger than the chloroplast genome (152,593 bp). However, compared to the chloroplast genes, the distribution of mitochondrial genes in *P. frutescens* is relatively sparse (Fig. 3 and Supplementary Table S3). In this study, based on sequence similarity between chloroplast and mitochondrial genomes, 70 chloroplast-like segments potentially involved in gene transfer were identified in the mitochondrial genome (Fig. 3). These inserted segments were distributed throughout the mitochondrial genome, with a total length of 12,680 bp, accounting for 4.23$\%$ of the entire mitochondrial genome. The longest sequence (875 bp) was transferred from the cp genome’s *psb*A gene to the intergenic regions of the mitochondrial genes *nad*2 and *atp*6, while the second longest sequence (820 bp) was transferred from the cp genome’s *pet*A gene to the intergenic regions of the mitochondrial genes *nad*2 and *atp*6. These transferred sequences were predominantly located in the mitochondrial genome’s intergenic spacers (27), ribosomal RNA genes (20), and transfer RNAs (19). In the *P. frutescens* chloroplast genome, 22 *rrn*16S and *rrn*23S sequences were inserted into the mitochondrial genome, with most of them being transferred to *rrn*18 or *rrn*26, except for 4 segments having been transferred to the IGS region. In the remaining chloroplast-like sequences, 15 *trn*A genes were completely transferred (*trn*P-UGG (2), *trn*F-GAA, *trn*S-GGA, *trn*T-GGU, *trn*D-GUC, *trn*S-GCU (2), *trn*Q-UUG, *trn*H-GUG, *trn*M-CAU (2), *trn*N-GUU, *trn*N-GUU, and *trn*W-CCA). Apart from *rps*11, which has been fully transferred to the mitochondrial genome *cox*1 and *rps*10’s IGS, the rest were partial sequences from the chloroplast genome (*psb*N, *psb*B, *psb*E, *pet*A, *acc*D, *rps*4, *ycf*3, *Psa*, *Psa*B, *psb*C, *rpo*B, *atp*A, *psb*A, *rpl*2, *rpl*23, *ycf*2, *nad*F, and *nad*H) transferred to partial genes or IGS of the mitochondrial genome.

### Condon usage and RSCU analysis

**Figure 4:**
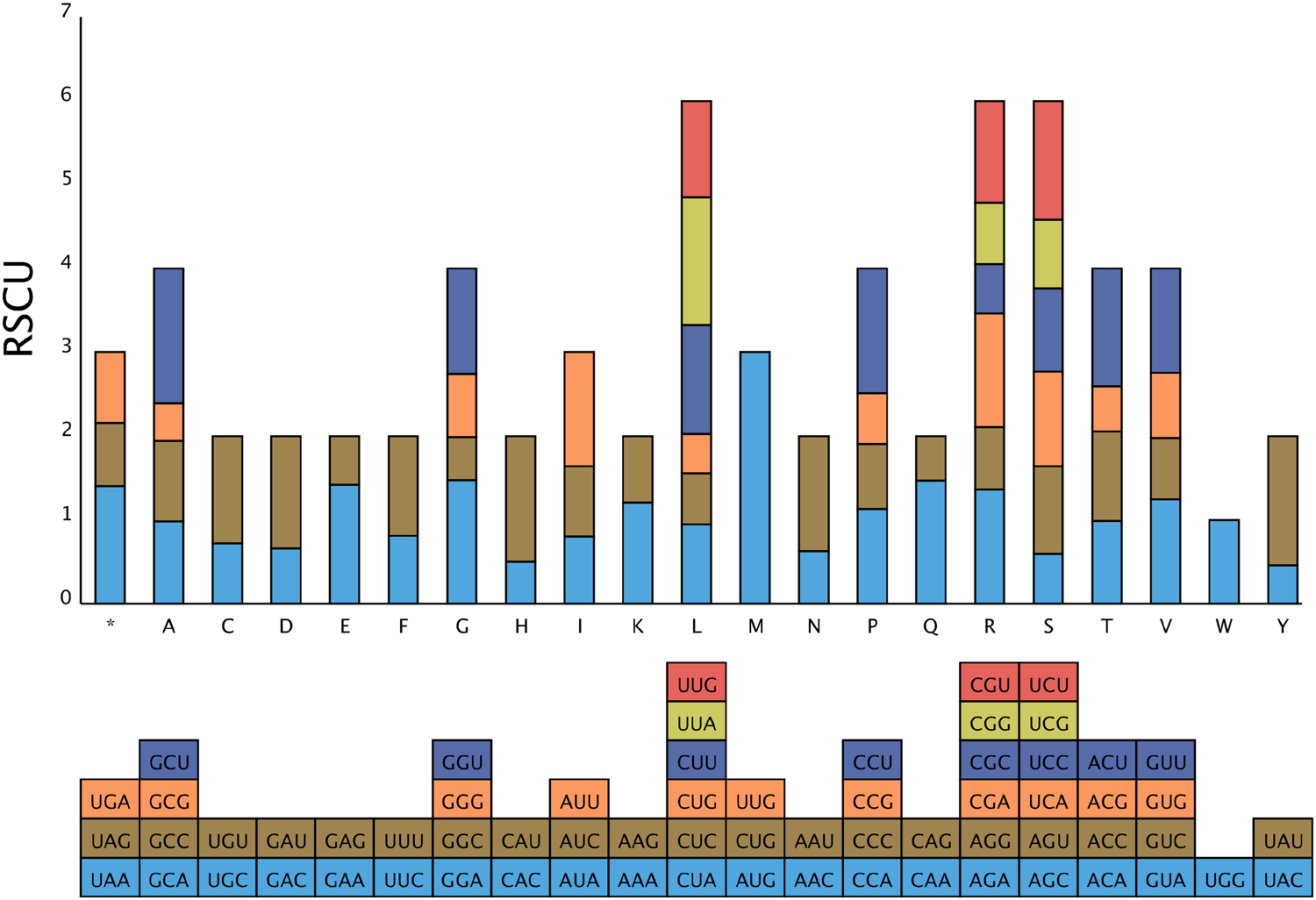
Analysis of relative synonymous codon usage (RSCU) in the *P. frutescen* mitogenome.

The total length of 34 PCGs in *P. frutescens* is 34,059 bp, containing a total of 9729 codons. The number of encoded codons varies from 8 to 389, with a total of 64 codons encoding 20 amino acids, including the stop codons (UAG (*), UAA (*), and UGA (*)). After excluding the three stop codons and the unbiased methionine (Met) and threonine (Thr), 31 codons among the 64 codons displayed a preference with RSCU values exceeding 1, indicating a higher priority for these codons. The GCU codon for Alanine (Ala) had the highest frequency of occurrence, with an average RSCU value of 1.61. The AUG codon coded for Methionine (Met) exhibited the highest frequency of occurrence, as indicated by an average RSCU value of 1.89. Among the analyzed codons, the remaining 31 codons displayed a relatively low bias, as indicated by their RSCU values less than 1 (Fig. 4). Meanwhile, results showed that codons ending with A or U had RSCU values mostly greater than 1.0, while those ending with C or G had RSCU values mostly less than 1. Additionally, codon usage was typically strongly biased towards A or T(U) at the third codon position, which was also observed in other plant mitochondrial genomes.

### Selective pressure analyses

**Figure 5:**
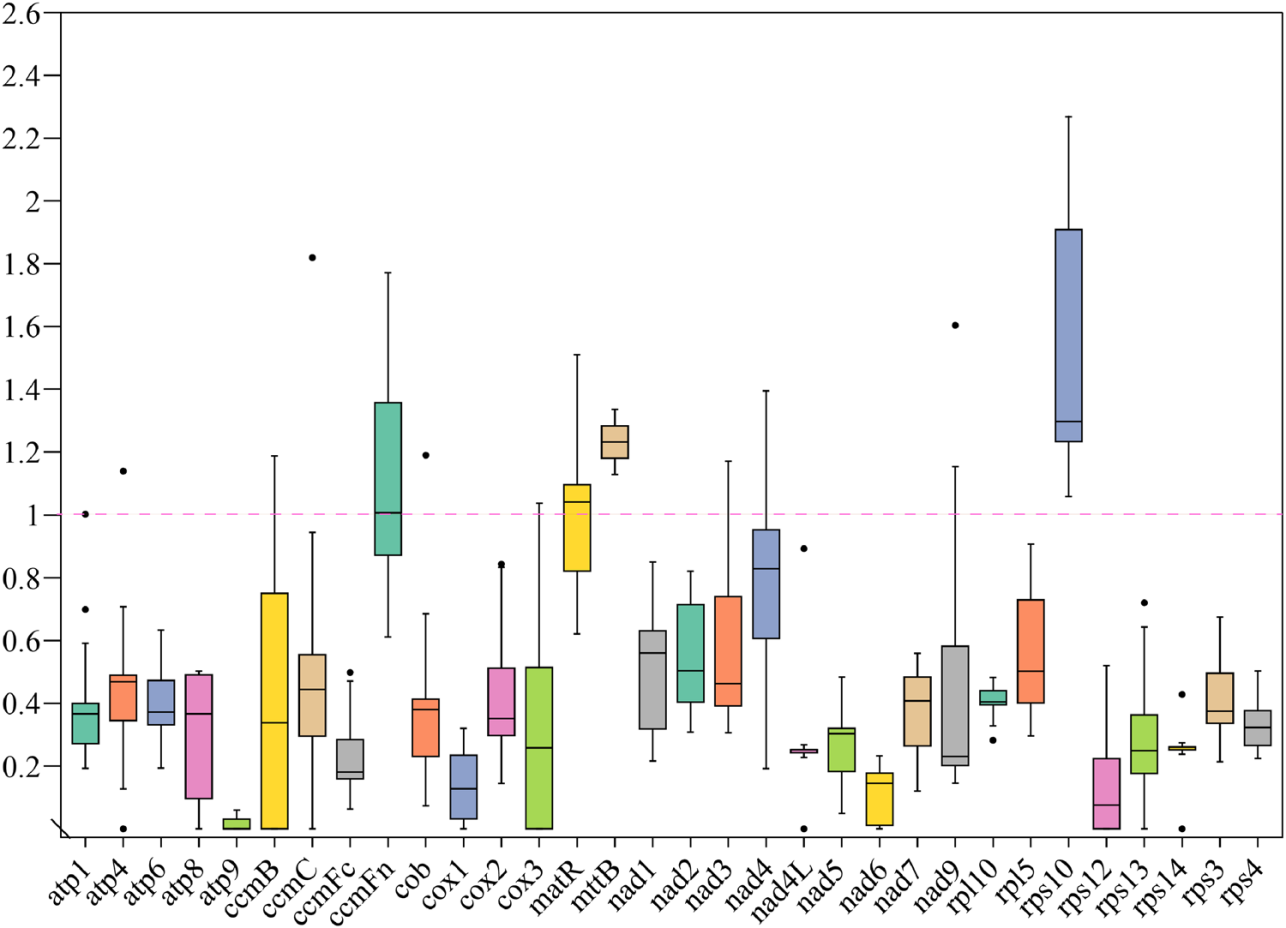
Boxplots of the Ka/Ks ratio of *P. frutescen* and 9 other plant species.

The Ka/Ks values of common protein-coding genes in the *P. frutescens* mitochondrial genome were calculated and compared with 9 Lamiaceae species (*Ajuga reptans*, *Lavandula angustifolia*, *Platostoma chinense*, *Pogostemon heyneanus*, *Prunella vulgaris*, *Rotheca serrata*, *Scutellaria barbata*, *Scutellaria franchetiana*, and * Vitex trifolia*) (Fig. 5). The average Ka/Ks value for 32 identical protein-coding genes was 0.33. Deeper investigation into the selective pressures on specific genes uncovered that except for *ccm*Fn, *mtt*B, *rps*10, and *mat*R showed Ka/Ks ratios > 1 compared to the other 9 plants, indicating that they might be undergoing positive selection. Most genes exhibited a negative selection effect (Ka/Ks < 1) compared to the other 9 plants, suggesting that most protein-coding genes in the *P. frutescens* mitochondrial genome were highly conserved during molecular evolution.

### RNA editing sites prediction

**Figure 6:**
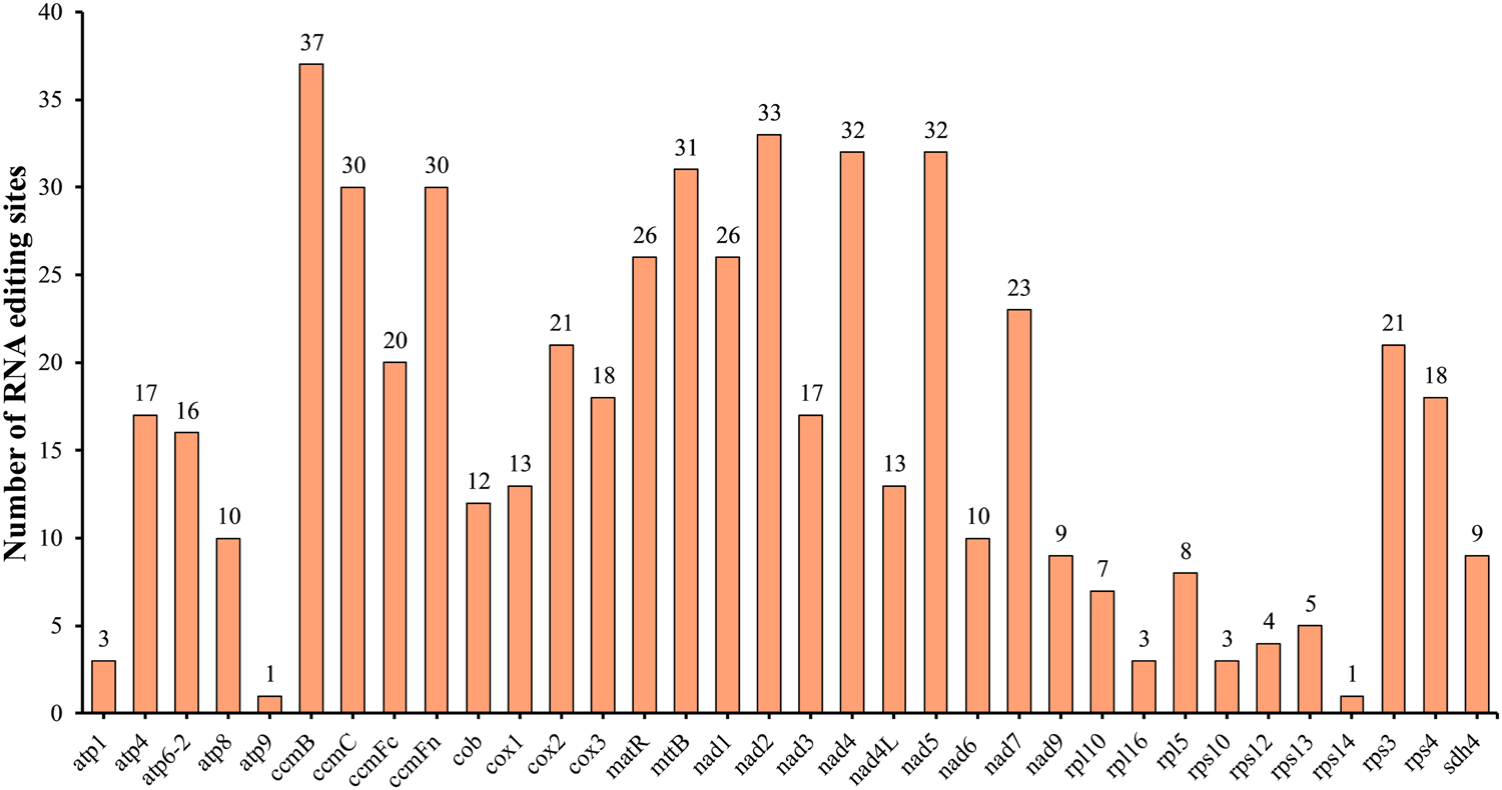
Distribution of RNA editing sites in the mt PCGs of *P. frutescens*.

RNA editing refers to the process of adding, removing, or substituting bases in the coding region of transcribed RNA, which occurs in all eukaryotes^15^. In the present study, a total of 559 RNA editing sites were predicted within the mitochondrial genome’s 34 protein-coding genes (PCGs) of *P. frutescen*. The distribution of these RNA editing sites varied among different genes, with the nad4 gene having the highest number at 48, followed by *ccm*B with 42 sites, making them the top two genes in terms of RNA editing sites (Fig. 6, Supplementary Table S4). Additionally, *mtt*B, *ccm*C, *nad*7, *nad*2, *ccm*FN, and *nad*5 each exhibited over 30 editing sites. Conversely, genes like *rps*14,* rpl*2, and *rps*7 presented the fewest editing sites, with only two C-to-U edits observed. Meanwhile, a total of 56 types of codon transitions were identified, yielding 25 different amino acid changes. Among these codon transition types, the UCA$\rightarrow$UUA transition occurred the most frequently, occurring at 64 sites. The translated amino acids were significantly affected by RNA editing events, with 558 RNA editing sites leading to non-synonymous substitutions and alterations in encoded amino acids, accounting for 99.83% of the total changes. Furthermore, the most common substitutions involved proline (Pro) and serine (Ser) being replaced by leucine (Leu) 100 and 99 times, respectively. Additionally, serine (Ser) was frequently substituted by phenylalanine (Phe) in 76 cases. Collectively, these three substitutions accounted for 49.23$\%$ of all observed changes. In comparison to the one synonymous substitution RNA editing event, these widespread RNA editing events held considerable significance in protein translation.

### Phylogenetic analysis

Reconstructing the phylogenetic tree based on conservative mitochondrial protein-coding genes is instrumental in elucidating the molecular evolutionary relationships among green plants. To determine the classification status of the *P. frutescens *mitochondrial genome, *P. frutescens* was hereby analyzed phylogenetically with 59 species, including 44 dicotyledonous plants (including 20 species of Lamiaceae), 13 monocotyledonous plants, 1 gymnosperm, and 1 bryophyte (outgroup). A total of 24 protein-coding genes corresponding to each species were also analyzed.

**Figure 7:**
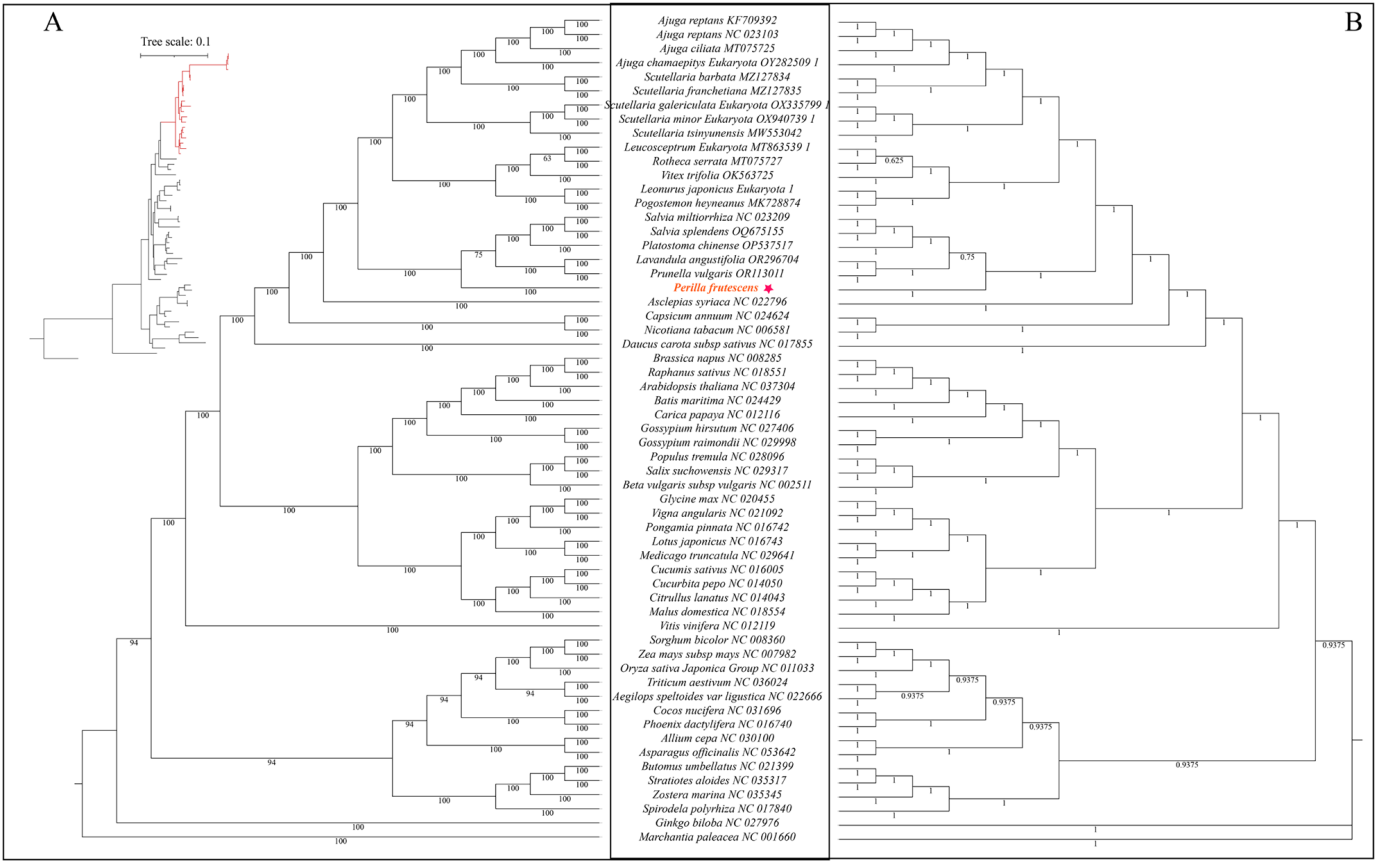
Phylogenies of 59 species inferred from whole mitogenomes. (**A**) Phylogeny of *P. frutescens *reconstructed by analyses of 24 concatenated plastid PCGs using maximum likelihood (ML) and Bayesian inference (BI) methods. Values at nodes represent the ultrafast bootstrap support values (UFBoot) of the maximum likelihood analysis and the posterior probabilities (PP) of the BI analysis values. (**B**) Species tree of *P. frutescens* reconstructed by ASTRAL based on 24 plastid protein-coding genes. Values at nodes represent local posterior probabilities (LPP).

Based on the concatenated dataset, both ML and BI phylogenies generated identical tree topologies, with nearly all nodes exhibiting high support values (ultrafast bootstrap (UFboot) = 100; SH-aLRT values (SH-aLRT) = 100; posterior probability (PP) = 1.00). The results indicated that the phylogenetic tree strongly supported the separation of dicots and monocots, as well as that of angiosperms and gymnosperms (Fig. 7). Furthermore, the clade distributions of Asparagales, Lamiales, Fabales, Brassicales, Alismatales, Poales, Cucurbitales, Gentianales, Malvales, Caryophyllales, Vitales, Rosales, Solanales, Apiales, Butomaceae, Zosteraceae, Marchantiophyta, Malpighiales, Ginkgoales and Arecales were well resolved in the phylogenetic tree. Relationships in the phylogenetic tree were found to be consistent with the traditional classification relationships of these species, indicating the congruence of traditional and molecular classifications at the family level. *P. frutescens* belonging to the Nepetoideae subfamily of the Lamiaceae family shared a highly similar topology to that of the phylogenetic reconstruction inferred from plastid genome and nuclear genome sequences. The species tree topology implemented in ASTRAL was basically congruent with the result of the concatenation analyses, with high local posterior probability (LPP) values at most nodes. Overall, the present analysis of the *P. frutescens* mitochondrial genome provides a valuable foundation for future studies on the phylogenetic relationships of species.

## Discussion

Mitochondria are essential organelles in eukaryotic cells. They are crucial for cellular respiration and energy metabolism, playing key roles in regulating important cellular activities such as differentiation, apoptosis, growth, and division^40,41^. The complex organization, diverse structures, dynamic non-coding sequences, and high levels of repetitive sequences in plant mitochondrial genomes all pose challenges in the study of plant mitochondria^4,15,42,43^. Herein, considering the much higher than copy number of plant organelle genomes compared to the corresponding nuclear genome, Illumina and PacBio sequencing technologies were combined to first describe the basic characteristics of the *P. frutescens *genome. The mitochondrial genome of *P. frutescens* was demonstrated as a circular structure with a total length of 299,551 bp. The genome sizes of Lamiaceae plants ranged from 271,618 to 729,504 bp, and the mitochondrial genome of *P. frutescens* was relatively small compared to other species in the Lamiaceae family. Its genome size was similar to the published mitochondrial genome of Prunella vulgaris (274,779 bp) in the Lamiaceae family^44^. The mitochondrial genome of *P. frutescens*, similar to most Lamiaceae plants, predominantly exhibited a circular structure, which might be attributed to the relative rarity of homologous recombination events, leading to structural conservation. However, some previously assembled mitochondrial genomes of Lamiaceae plants exhibited multi-chromosomal or multi-branch structures. For instance, *Ajuga decumbens* (374,491 bp) and *Teucrium ornatum* (608,646 bp) each had up to 8 and 9 isolated scaffolds, respectively. The occurrence of such diverse chromosomal structures in mitochondrial genomes within the same family highlighted the evolutionary adaptability and complexity of plant mitochondrial genomes^45^. Nevertheless, The mitochondrial genome of angiosperms averages 475 kb, and the mitochondrial genome of Lamiaceae has a smaller and more conserved structure compared to other angiosperms? Besides, the depth and length of sequencing data during the assembly process also significantly impacted the assembly results^4^, warranting further investigation upon the mitochondrial genome structures of *Ajuga decumbens* and *Teucrium ornatum*. The GC content of the mitotic genome of *P. frutescens* was 45.23$\%$, which was highest in the rRNA genes. The result was consistent with other Lamiaceae species such as *Scutellaria tsinyunensis* (45.09$\%$)^46^, *Rotheca serrata* (45.53%)^47^, and *P. vulgaris *(43.92%)^44^. This suggested that the GC content of the mitotic genome was relatively conserved during the evolutionary process in plants. In angiosperms, the mitotic genome encoded 24 core protein-coding genes, including *atp*1,* atp*4, *atp*6, *atp*8, *atp*9, *ccm*B, *ccm*C, *ccm*Fc, *ccm*Fn, *cob*, *cox*1, *cox*2, *cox*3, *mat*R, *mtt*B, *nad*1, *nad*2, *nad*3, *nad*4, *nad*4L, *nad*5, *nad*6, *nad*7, and *nad*9, most being respiratory protein genes^48^. In the mitochondrial genome of *P. frutescens*, a total of 59 genes were identified, including 37 protein-coding genes (26 were core genes), 20 tRNA genes, and 2 rRNA genes. The types and quantities of these genes were consistent with the core mitochondrial genes of most Lamiaceae plants (Supplementary Table S5)^44,46,49^. The coding region of the *P. frutescens* mitochondrial genome accounted for only 19.23$\%$ of its total length, with over 80$\%$ being non-coding regions. This might result from the gradual increase of repeat sequences in the mitochondrial genome during evolution^47^.

The calculation of non-synonymous substitutions (Ka) and synonymous substitutions (Ks) plays a critical role in phylogenetic reconstruction and understanding the evolutionary dynamics of protein-coding sequences among closely related species^44^. In genetics, the Ka/Ks ratio serves as a key indicator to determine whether specific protein-coding genes are subject to selection pressure during evolution. A Ks value equaling Ka, or the Ka/Ks ratio equaling 1 suggests neutral selection. A Ka/Ks ratio greater than 1 indicates positive selection, where the non-synonymous substitution rate (Ka) exceeds the synonymous substitution rate (Ks). Conversely, negative selection appears if Ks surpasses Ka or the Ka/Ks ratio is less than 1^50^. Herein, few protein-coding genes were affected by positive selection, aligning with existing research reports^3,43,46,51,52^. The Ka/Ks analysis of the mitochondrial genome indicated that widely present PCGs (protein-coding genes) in mitochondrial were retained. However, genes with Ka/Ks > 1, such as *ccm*Fn, *mtt*B, *rps*10, and *mat*R were also detected, indicating that positive selection played a role in the evolutionary history of these coding genes. Besides, high Ka/Ks genes were considered important in the study of gene selection and evolution in Lamiaceae plants.

The transfer of genes between chloroplast and mitochondrial genomes is an important feature of plant mitochondrial genome evolution^53^. When exogenous genes are inserted into the mitotic genome, they are preferentially inserted into intergenic regions^54,55^. This phenomenon is a major reason for the observed differences in the number of coding genes in mitochondrial genomes among different plant species. Therefore, tracking gene transfer matters considerably in exploring the evolution of plant mitochondrial genomes. The length of chloroplast genome-integrated DNA into the mitotic genome varies among plant species, but generally falls within the range of 1–12$\%$ of the angiosperm plastome sequence^56^, such as in *Garcinia mangostana* L. (1.7$\%$)^57^ and *cucurbita pepo* (11.6$\%$)^58^. In this study, 70 migrating segments with a total length of 12,680 bp were identified, accounting for 4.23$\%$ of the entire mitochondrial genome. In the evolutionary history of mitochondrial genomes, widespread loss events of tRNA were observed, and the lost tRNAs in mitochondrial genomes could be replaced by tRNAs from other organelles^59^. In this study, it was discovered that the chloroplast rps11 and 15 tRNA genes completely transferred to the Mitochondrial genome, a common phenomenon in plants featuring frequent transfer of tRNA from chloroplasts to mitochondria^60^, and that chloroplast-derived tRNAs might have potential functional complementation. Furthermore, a significant amount of chloroplast gene fragments migrating to the mitochondrial genome were also identified. These chloroplast-derived fragments contained genes playing important roles in chloroplast function. However, their functionality in the mitochondrial genome remains unclear in this study. Two possible explanations were proposed for the fate of exogenous genes in the mitochondrial genome: (1) Transferred genes typically lost their functionality, while naturally functional copies coexisted in the mitochondrial genome^61^; (2) Natural genes were lost from the mitochondrial genome, and exogenous copies might have a function in maintaining normal cellular operations^62^. Hence, understanding the patterns of sequence transfer plays an important role in tracing ancient recombination events and structural variations in plant mitochondrial genomes, requiring more attention.

In plants, RNA editing enzymes catalyze conversion of specific nucleotide positions in mitochondrial RNA sequences from C to U or U to C. RNA editing holds much significance in regulating mitochondrial gene expression and function by inducing alterations in RNA sequences, thereby influencing the translated protein products^63–65^. RNA editing is an important source of variation in angiosperm mitochondrial genomes^66^. Additionally, the mechanism of crop male sterility has been associated with RNA editing processes. Herein, in the mitochondrial genome of *P. frutescens*, 559 RNA editing sites were identified, primarily occurring at the first and second positions of codons, with the editing method being cytosine <=> uracil (C <=> U). The preference for G/C codons might be attributed to their high binding energy, which played a role in maintaining translation accuracy^67^. By identifying RNA editing sites, researchers could gain valuable insights into the functional implications of predicting newly encoded codons. Notably, RNA editing events produced stop codons in the *rps*10 gene, and start codons in three genes (*cox*2, *nad*4L, and *rps*10), which could often be related to the production of highly conserved and homologous proteins found in other species, thereby promoting efficient gene expression in mitochondria. Furthermore, RNA editing events were found to be least common in ribosomal protein genes like *rps*14, while genes like *nad*5, *mtt*B, and *ccm*B exhibited higher frequencies of RNA editing events. These findings suggested the crucial role of RNA editing in plant adaptation to environmental changes and signal transduction^68,69^. Codon usage bias is an important factor reflecting the evolution of mitochondrial genomes^70^. Generally, factors such as mutation, natural selection, and phylogenetic relationships may lead to differences in codon usage preferences^71^. In the mitochondrial genome of *P. frutescens*, 30 codons with bias (RSCU > 1) were hereby identified, and these codons used more A/T bases. Therefore, upcoming research should account for codon usage biases influenced by mitochondrial RNA editing sites in the development of male sterile materials, thereby facilitating advancements in genetic breeding programs.

Repeat sequences are widely present in mitochondrial genomes and are usually crucial for molecular recombination, structural variation, and extreme differences in mitotic genome size^72^. These sequences also serve as important sources of information for population development and evolutionary analysis markers^73^. In this study, a total of 293 repeats, including 141 pairs of reverse complementary repeats and 152 pairs of forward repeats, were observed, possibly revealing frequent molecular recombination in mitochondrial genomes. This frequent recombination could play a crucial role in dynamically changing the structure and conformation of the mitochondrial genome during the evolutionary process. Simple sequence repeats (SSRs) represent a distinctive class of repetitive DNA sequences characterized by high repetitiveness, extensive polymorphism, and frequent co-dominance, and are abundantly dispersed throughout plant genomes^38^. SSR markers provide useful information for analyzing genetic diversity, genetic relationships, and population structures in crop species germplasm. In the present study, 77 SSRs were distributed in different genomic regions of *P. frutescens* mitotic genomes. The most abundant SSR type was tetrameric repeats (27.35$\%$), followed by dimeric repeats (20$\%$), consistent with previous studies on mitochondrial genomes. Moreover, a nearly universal A/T bias was also observed in the mitochondrial genome of *P. frutescens*. These SSRs consisted of motifs rich in A and T, also consistent with previous observations. The findings further confirmed the correlation between the AT content of the complete mitotic genome and SSRs. Extensive collection of genetic resources in *P. frutescens* could be found^74^. While numerous plant genetic resources have been collected in gene banks worldwide, the large sample size and lack of sufficient information on population structure and genetic diversity still terribly hinder the successful utilization of the genetic potential of plant genetic resources^25,74,75^. Currently, efforts have shifted from the collection of plant genetic resources to the identification of genetic diversity and core germplasm in *P. frutescens*^74,76–78^. The identified SSR sequences will provide useful information for the effective protection and utilization of genetic resources and the selection of useful genetic resources for* P. frutescens *breeding programs.

## Methods

### Plant sampling, DNA extracting and sequencing

In December 2023, fresh leaves of *P. frutescens* were collected at the Baiyun Experimental Base of Guangdong Academy of Agricultural Sciences (N23$^\circ$07$\prime$03$\prime \prime$, E113$^\circ$08$\prime$36$\prime \prime$). The plant had been grown for 6 months. The collected leaves were immediately cooled with liquid nitrogen and stored in an ultra-low temperature freezer at $-80\; ^{\circ }$C. Total DNA was extracted using a plant genomic DNA kit (TSINGKE Biotech, Beijing, China), and the quality and quantity of the extracted DNA were assessed using a NanoDrop One spectrophotometer (Thermo Fisher Scientific, Massachusetts, USA) and the Qubit dsDNA HS assay kit on a Qubit 3.0 fluorometer (TSINGKE Biotech, Beijing, China), as well as agarose gel electrophoresis. Subsequently, the extracted DNA samples were shipped in dry ice to Wuhan BMK Tech Solutions Company Limited (Qingdao, China) for SMRT and Illumina sequencing.

### Genome assembly and annotation

The raw read data were filtered and corrected using the Pacbio RS II sequencing technology, while sequencing adapters and low-quality sequences were filtered out using the SMRT Analysis (v2.3.0) with default settings to obtain clean reads. Then, the mt genome sequence of *P. frutescens* was extracted from the filtered reads containing chloroplast and mitochondrial genomes. Besides, the NCBI chloroplast genome data were used for BLAST^79^ filtering of reads containing chloroplast genomes, and reads with a match percentage greater than 90% were removed. Following that, the third-generation assembly software Canu^80^ was employed to correct the obtained third-generation data, and Bowtie2 (v2.3.5.1) was adopted to align the second-generation data with the corrected sequences. Using default parameters, Unicycler (v0.4.8)^81^ concatenated the aforementioned second-generation data and the corrected third-generation data, yielding a circular mitochondrial genome of *P. frutescens*. The average depth of the assembled mitochondrial genome was 152.25$\times$ (Supplementary Table S6). Furthermore, for mitochondrial genome annotation, the following steps were performed: BLAST was used to compare encoded proteins and rRNA with published plant mitochondrial sequences, followed by manual adjustments based on closely related species. tRNA annotation was conducted using tRNAscan-SE with default settings (http://lowelab.ucsc.edu/tRNAscan-SE/)^82^. ORFs were annotated using Open Reading Frame Finder (http://www.ncbi.nlm.nih.gov/gorf/gorf.html). Besides, the circular mitochondrial genome map was visualized using the online software Draw Cellelle Genome Map^83^. Following these steps, the assembly and annotation of the *P.frutescens* mitochondrial genome could be accomplished, providing essential foundational data for further research.

### Repeat sequence analysis

Three types of repeat sequences (simple, tandem, and dispersed) were detected in the mitochondrial genome. Simple repeat sequence analysis was performed using the online software MISA (https://webblast.ipk-gatersleben.de/misa/)44. In this analysis, 10, 5, 4, 3, 3, 3, and 3 repeat sequences were identified with 1, 2, 3, 4, 5, and 6 base pairs, respectively. Tandem repeat sequences with a length exceeding 6 bp and a repeat unit match of over 95 % were detected using the online tool Tandem Repeats Finder v4.09 (http://tandem.bu.edu/trf/trf.submit.options.html)^84^. The following parameters were involved: 2 7 7 80 10 50 2000 -f -d -m. Dispersed repeat sequences were identified using BLASTN (v2.10.1) with parameters of word size 7, e-value 1e-5. Redundancy, and tandem repeats were removed. Circos v0.69-5 (http://circos.ca/software/download/)^85^ was used for visualizing these repeat sequences. Thorough repeat sequence analyses could provide valuable insights into the repeat elements in the *P. frutescens* mitochondrial genome, illuminating its genomic organization and evolution.

### Exploration of chloroplast to mitochondrion DNA transformation, RNA editing, codon usage patterns, and selection pressure

In this investigation, an online cloud platform was utilized to analyze the codon composition of the *P. frutescens* mitochondrial genome, screen for unique CDS, and determine the relative synonymous codon usage (RSCU) of each gene. The chloroplast genome sequence of *P. frutescens* was obtained from the NCBI Organelle Genome Resources database. By employing the BLAST software on NCBI, homologous fragments between the mitochondrial and chloroplast genomes were identified, and the screening criteria were set at match rate $\ge$ 70$\%$, E-value $\le$ 1e−5, and length $\ge$30 bp. The results were visualized using Circos (v0.69-5). The Deepred-mt was used for the prediction of RNA editing of the mitochondrial genomes. Furthermore, genbank files of 9 Lamiaceae species were imported into the Ka/Ks cloud tool to calculate Ka/Ks values for 32 shared proteins. Subsequently, one-way analysis of variance on the Ka/Ks ratios of the 32 protein-coding genes was conducted using the R programming language.

### Phylogenetic inference

A phylogenetic tree was plotted using the mitochondrial genomes of 50 species downloaded from NCBI, with *Marchantia paleacea* taken as the outgroup. To ensure comparability, these mitochondrial genomes were re-annotated using the previously described tools. PhyloSuite (v.1.2.1)^86^ was utilized to identify and extract 30 orthologous mitochondrial genes from the analyzed species. Besides, the corresponding nucleotide sequences were aligned using MAFFT (v.7.450)^87^. Subsequently, these aligned sequences were concatenated and utilized to construct the phylogenetic tree. ModelFind was used to build the best model with default parameters, and the maximum likelihood (ML) analysis with 1000 bootstrap replicates was performed in RAxML (v.8.2.4)^88^. Furthermore, Bayesian inference (BI) analysis was conducted using MrBayes (v.3.2.6)^89^. The Markov chain Monte Carlo method was adopted for 200,000 generations, with tree sampling conducted every 1000 generatiotabns for precision. The first 20$\%$ of trees were discarded as burn-in, while the remaining were used to generate the consensus tree. For the ASTRAL^90^analysis, 24 mitochondrial gene trees were inferred separately using RAxML , utilizing the GTRGAMMA model alongside 100 bootstrap replicates. To enhance the accuracy of species-tree inference, branches (<10$\%$ bootstrap support) within these gene trees were pruned subsequently, the collapsed gene trees were imported into ASTRAL-III with the default settings. This process yielded an estimated topology of the species tree with branch lengths and local posterior probabilities (LPP) as branch support values.

### Statement of plant collection

We hereby declare that *Perilla frutescens* is not a plant species covered by the IUCN Policy Statement on Research Involving Species at Risk of Extinction and the Convention on the Trade in Endangered Species of Wild Fauna and Flora. The botanical collection work involved in this research has obtained the necessary permits and approvals from relevant local institutions, and strict compliance with applicable laws and guidelines has been ensured. Moreover, we have minimized the impact on the environment and ecosystems during the collection process, and made every effort to maintain the survival and reproductive capacity of the *P. frutescens* plants.

## Conclusions

In this research, the sequencing and assembly of the mitochondrial genome of *P. frutescens* were successfully completed, shedding light on a comprehensive comparison of the organelle genome. This breakthrough is anticipated to provide a broader perspective for the investigation of gene transfer between mitochondrial and plastids. Additionally, through phylogenetic analysis of the mitochondrial genomes of this species and 50 other taxa, the evolutionary status of *P. frutescens* was definitively determined. Overall, the findings offer valuable insights to lay the groundwork for future research on genetic variation, systematic evolution, and breeding of *P. frutescens*, thereby facilitating the cultivation, development, and utilization of this valuable plant.

## Supplementary Information

Supplementary Tables.

## Data Availability

The complete mitochondrial genome of *P. frutescens* generated in this study was submitted to the NGDC database (https://ngdc.cncb.ac.cn/genbase/search/gb/C_AA059311.1) with GeneBase accession number C_AA059311.1. The associated BioProject are PRJNA1108956.
